# Focal Targeting of the Bacterial Envelope by Antimicrobial Peptides

**DOI:** 10.3389/fcell.2016.00055

**Published:** 2016-06-07

**Authors:** Rafi Rashid, Mark Veleba, Kimberly A. Kline

**Affiliations:** Singapore Centre for Environmental Life Sciences Engineering, School of Biological Sciences, Nanyang Technological UniversitySingapore, Singapore

**Keywords:** cationic antimicrobial peptide, focal targeting, membrane lipid homeostasis, antimicrobial peptide resistance, antimicrobial pepetide sensing

## Abstract

Antimicrobial peptides (AMPs) are utilized by both eukaryotic and prokaryotic organisms. AMPs such as the human beta defensins, human neutrophil peptides, human cathelicidin, and many bacterial bacteriocins are cationic and capable of binding to anionic regions of the bacterial surface. Cationic AMPs (CAMPs) target anionic lipids [e.g., phosphatidylglycerol (PG) and cardiolipins (CL)] in the cell membrane and anionic components [e.g., lipopolysaccharide (LPS) and lipoteichoic acid (LTA)] of the cell envelope. Bacteria have evolved mechanisms to modify these same targets in order to resist CAMP killing, e.g., lysinylation of PG to yield cationic lysyl-PG and alanylation of LTA. Since CAMPs offer a promising therapeutic alternative to conventional antibiotics, which are becoming less effective due to rapidly emerging antibiotic resistance, there is a strong need to improve our understanding about the AMP mechanism of action. Recent literature suggests that AMPs often interact with the bacterial cell envelope at discrete foci. Here we review recent AMP literature, with an emphasis on focal interactions with bacteria, including (1) CAMP disruption mechanisms, (2) delocalization of membrane proteins and lipids by CAMPs, and (3) CAMP sensing systems and resistance mechanisms. We conclude with new approaches for studying the bacterial membrane, e.g., lipidomics, high resolution imaging, and non-detergent-based membrane domain extraction.

## Introduction

Bacterial infections are a major concern in hospitals where the mortality rate and duration of hospital stay are up to double for patients with drug-resistant vs. drug-susceptible infections (Holmberg et al., 1987; Carmeli et al., 2002; Cosgrove et al., 2002), and the economic burden of hospitalization is correspondingly higher (Smith et al., 2004). While selection pressure can lead to antibiotic resistance among many bacterial species, of particular concern are the group of bacteria known as the “ESKAPE” pathogens (Rice, 2008), which include *Enterococcus faecium, Staphylococcus aureus, Klebsiella pneumoniae, Acinetobacter baumanii, Pseudomonas aeruginosa*, and *Enterobacter* spp., all of which are commonly antibiotic resistant and significantly complicate treatment procedures (Rice, 2010). With decreasing antibiotic effectiveness in resistant strains, investigations into antimicrobial peptides (AMPs) as an alternative form of therapy are of interest, as they have unique mechanisms of action and have not been widely used as conventional antibiotics. As advances in biotechnology enable improved AMP synthesis, it is possible that AMPs will emerge as promising alternatives to conventional antibiotics (Hancock and Sahl, 2006) owing to their relatively simple methods of synthesis and modification. Additionally AMPs have already been characterized as effective against ESKAPE strains resistant to conventional antibiotic treatments (Wu et al., 2011; Menousek et al., 2012; Vila-Farres et al., 2012).

AMPs are found in virtually all domains of life including eukaryotes, archaea, and bacteria (Jenssen et al., 2006; Zhao et al., 2013). Bacteriocins, bacteria-derived antimicrobial peptides, can be subdivided into several groups. In Gram-negative bacteria, most bacteriocins belong to either the microcins (relatively small, post-translationally modified peptides) and the larger colicins (reviewed by Cascales et al., 2007; Duquesne et al., 2007, respectively). Gram-positive-derived bacteriocins, primarily produced by lactic acid bacteria, can broadly be divided into two classes: lantibiotics and non-lantibiotics (Papagianni, 2003). Nisin, a well-studied lantibiotic, is already widely used in food preservation (Delvesbroughton, 1990; Hansen, 1994).

In humans, cationic antimicrobial peptides (CAMPs) are released by host epithelial and immune cells (e.g., neutrophils and macrophages) during infection and are part of the innate immune response against pathogens. The mammalian immune system produces two main classes of CAMP: (1) the defensins, which include human neutrophil peptides (HNP) and human β-defensins (HBD); and (2) the cathelicidins, of which LL-37 is the only representative found in humans (Durr et al., 2006). CAMPs are amphipathic molecules: one portion of the molecule is positively charged and attracted to the negatively-charged phospholipid head groups in the bacterial membrane, and the other portion is hydrophobic and capable of inserting itself into hydrophobic membrane regions (i.e., the fatty acid chains). The outer leaflet of the mammalian cell membrane contains zwitterionic phospholipids like phosphatidylcholine (PC) and phosphatidylinositol (PI), whereas the bacterial membrane contains anionic phospholipids like phosphatidylserine (PS), phosphatidylglycerol (PG), and di-phosphatidylglycerol (D-PG), also known as cardiolipin (CL), as well as the zwitterionic lipid phosphatidylethanolamine (PE) (van Meer and de Kroon, 2011).

In this review, we explore how CAMPs focally target bacterial cells to disrupt lipid domains and localized proteins in the bacterial membrane. We begin with a brief overview of CAMP membrane disruption mechanisms, specifically focusing on the resulting delocalization of membrane proteins and lipids. The bacterial membrane also plays a crucial role in cellular homeostasis, adapting to environmental changes, including exposure to cationic antimicrobials, either from innate immune sources or therapeutic intervention. Antimicrobial sensing and response systems often lead to altered transcription of resistance-associated genes and we will therefore subsequently explore CAMP resistance mechanisms in various bacterial species. We conclude the review with suggestions for future research and the experimental techniques that are needed for realizing these goals.

## CAMP membrane disruption mechanisms

CAMPs generally rely on membrane disruption to cause bacterial cell death. To date, three models of CAMP membrane disruption have been proposed: the barrel-stave model, the toroidal model, and the carpet model.

In the barrel-stave model, AMPs insert and diffuse laterally through the lipid bilayer (Ehrenstein and Lecar, 1977), arranging into helices, and creating barrel/stave-like channels that span the membrane (Matsuzaki et al., 1991). Hydrophobic regions of the CAMP face the lipid bilayer while hydrophilic regions face the pore lumen (Baumann and Mueller, 1974). This model has been suggested for fungal CAMP alamethicin, pardaxin (from the Red Sea sole), cecropins (isolated from moths) and, initially, magainins (from frogs) (Christensen et al., 1988; He et al., 1996; Matsuzaki, 1998; Porcelli et al., 2004).

In the toroidal model, the peptide molecules maintain a predominantly parallel orientation to the membrane (Leontiadou et al., 2006). A water core is formed in the center of the pore, with the AMPs and lipid head groups forming the wall of the pore (Ludtke et al., 1996; Matsuzaki et al., 1996), while the lipid monolayers bend back on themselves continuously outside the pore (Ludtke et al., 1996; Matsuzaki et al., 1996; Yang et al., 2001). Magainins (from frogs), melittin (from bee venom), and protegrins (from porcine leukocytes) all follow this mode of action (Yang et al., 2001; Leontiadou et al., 2006; Tang and Hong, 2009).

In the carpet model, the peptides do not form pores but bind parallel to the membrane surface, forming a “carpet” in association with other peptide monomers (Pouny et al., 1992). At a certain concentration of peptide, the bilayers are disrupted and form micelles, destroying the membrane structure in a detergent-like manner (Pouny et al., 1992; Hancock, 1997). LL-37 has been associated with this mechanism (Porcelli et al., 2008).

## Disruption via delocalization of membrane lipids and proteins

It is becoming increasingly clear that the initial point of interaction between many CAMPs and the cell surface occurs at discrete foci. The consequences of this focal interaction can include the delocalization of similarly focally localized membrane proteins and lipids domains. The lantibiotic family of AMPs kills bacteria through pore formation (Brötz et al., 1998; Breukink et al., 1999), but Hasper et al., proposed an updated model of killing involving focally targeted membrane disruption for the lantibiotic nisin. Nisin's target is lipid II, a constituent of the cell wall biosynthetic machinery (Breukink and de Kruijff, 2006). Nisin binds to and causes lipid II to segregate in the membrane of giant unilamellar vesicles (GUVs) and disrupts the septal and helical distribution pattern of lipid II in *Bacillus* cells (Hasper et al., 2006). In GUVs, nisin caused lipid II to segregate, forming patches in the membrane. When added to *Bacillus subtilis* and *Bacillus megaterium* cells, nisin caused fluorescent vancomycin-labeled lipid II to shift from the division septum to other parts of the membrane (Hasper et al., 2006). In a more recent study, treating GUVs with fluorescently-labeled nisin resulted in the formation of large aggregates, which were visible as bright patches in the membrane. Through dual-color fluorescence imaging, these patches were shown to contain nisin and lipid II. The size of these aggregates determined the efficiency of membrane permeation by nisin (Scherer et al., 2013). The presence of nisin aggregates was recently shown to coincide with membrane damage and cell death and to cause GUV shrinkage via vesicle budding, suggesting that nisin aggregation causes destructive membrane deformation leading to cell killing. Nisin aggregates had to reach a certain size for cell death to occur (Scherer et al., 2015).

Other CAMPs similarly interact with the cell membrane at discrete foci, possibly at membrane microdomains. These domains are involved in bacterial cell division, cell differentiation, and protein secretion, and have been reviewed elsewhere (Epand and Epand, 2009; Barák and Muchová, 2013; Bramkamp and Lopez, 2015). At sublethal concentrations, the human neutrophil peptide 1 (HNP-1), and polymyxin B (PxB), an antibiotic with cationic detergent-like action preferentially target an anionic lipid microdomain that coordinates the localized secretion of virulence-associated proteins in *Streptococcus pyogenes.* At higher concentrations that were still sublethal, the same peptides disrupted the microdomain and delocalized its associated proteins involved in protein secretion (e.g., the SecA ATPase; Vega and Caparon, 2012). Similarly, sub-inhibitory concentrations of the CAMP human β-defensin 2 (hBD2) targeted the surface of *Enterococcus faecalis* in a focal manner, and simultaneously disrupted the localization of SecA as well as sortase A (SrtA), an enzyme required for the assembly and covalent attachment of virulence-associated proteins to the cell wall. The anionic lipid stain, nonyl acridine orange (NAO), stained the *E. faecalis* membrane focally, suggesting that focal CAMP binding may occur at these discrete anionic lipid domains (Kandaswamy et al., 2013). Taken together, these observations suggest a model in which localized virulence factor secretion and assembly sites colocalize with anionic lipids, which can be targeted by CAMPs.

Similarly, recent studies of sublethal concentrations of a six-amino-acid cationic peptide RWRWRW-NH_2_ (MP196 for short), consisting of alternating arginine and tryptophan residues, demonstrated that interaction with *B. subtilis* resulted in delocalization of the peripheral membrane proteins MurG and cytochrome c (Wenzel et al., 2014). MurG is an enzyme that converts lipid I to lipid II and cytochrome c is involved in energy metabolism. Delocalization of these proteins was independent of membrane potential changes. Thus, the authors proposed that delocalization was likely caused by changes in membrane architecture resulting from MP196 integration, which would be facilitated by the lipophilic tryptophan residues and promote interactions between the cationic arginine residues and negative phospholipid head groups (Wenzel et al., 2014). Furthermore, the addition of lysine to MP196 followed by lipidation with a C_8_-acyl chain resulted in enhanced antimicrobial activity against *B. subtilis* without any change in the mechanism of action (Wenzel et al., 2015). For other peptides, lipidation was shown to enhance interactions with the lipid bilayer (Zweytick et al., 2011; Nasompag et al., 2015). In a separate study, *Escherichia coli* cells treated with N-acylated AMPs derived from lactoferricin showed disruption of phosphatidylethanolamine (PE) and cardiolipin domains, which resulted in defective cell division followed by cell death (Zweytick et al., 2014).

When *E. coli* cells were treated with the CAMP Cecroprin A, high time-resolution fluorescence microscopy showed that permeabilization of the outer and cytoplasmic membranes occurred in discrete membrane regions. This localized membrane disruption was persistent and stable over time. At a concentration four times the minimum inhibitory concentration (MIC), Cecroprin A bound to the membranes of dividing cells (at the septum, where cell wall synthesis occurs) earlier than it bound to nonseptating cells (at the cell poles, where there is no cell wall synthesis; Rangarajan et al., 2013). Both the septa and poles of *E. coli* are rich in anionic phospholipids such as cardiolipins (Mileykovskaya and Dowhan, 2009). These data suggest that septal and/or polar curvature, promoted by CL, may be responsible for the localized membrane disruption by Cecropin A (Rangarajan et al., 2013).

Another cationic antimicrobial agent is the lipopeptide antibiotic daptomycin, which is thought to interact with negatively charged membrane lipids such as PG and/or CL. Reduced daptomycin susceptibility in *S. aureus* strains is linked to mutations in multiple peptide resistance factor *(mprF).* MprF synthesizes lysyl-PG, a modified cationic version of PG, changing the membrane charge from negative to positive, and limiting the ability of daptomycin to bind to the membrane in a calcium-dependent manner (Friedman et al., 2006; Ernst et al., 2009; Zhang et al., 2014). Gain-of-function *mprF* mutations, which increase levels of outer membrane leaflet lysyl-PG and/or MprF synthesis, in daptomycin-resistant *S. aureus* further limit daptomycin binding to the cells (Jones et al., 2008; Mishra et al., 2009; Yang et al., 2010). In *E. faecalis*, daptomycin at inhibitory concentrations targets the cell at the division septum, and daptomycin-resistance correlates with the failure of daptomycin to bind focally to the division septum (Tran et al., 2013). Altered daptomycin targeting in daptomycin-resistant *E. faecalis* was associated with a point mutation in *liaF* that changed the distribution of CL microdomains, thus preventing daptomycin from binding to the division septum. LiaF is a transmembrane protein that is a member of the three-component regulatory system LiaFSR responsible for cell envelope homeostasis (Tran et al., 2013). In *B. subtilis*, daptomycin also binds focally to membrane regions rich in PG resulting in membrane distortions (Hachmann et al., 2009). DivIVA mislocalization as a result of these distortions leads to aberrant cell morphology, membrane rupture, and cell death (Pogliano et al., 2012). DivIVA mislocalization may be explained by the observation that DivIVA recognizes and binds to regions of high curvature (Lenarcic et al., 2009), which arise upon daptomycin exposure. This model would also explain the morphological aberrations seen in *S. aureus* after treatment with sub-MIC daptomycin (Cotroneo et al., 2008).

Taken together, it is becoming clear that a variety of cationic antimicrobial agents preferentially interact with the bacterial cell at discrete domains, resulting in the dispersion of those domains and often in the disruption of functions governed by those domains. Table 1 summarizes recent literature reporting focal interactions between AMPs and bacteria. A deeper understanding of bacterial membrane composition and organization will enable further elucidation of the relationship between lipids, membrane domains, focal CAMP targeting, and CAMP killing.

**Table 1:** **Studies reporting focal interactions between antimicrobial peptides and bacteria**.

**Authors**	**Species**	**Findings**
Hasper et al., 2006	*Bacillus subtilis* and *Bacillus megaterium*	Nisin disrupted septal and helical distribution of lipid II and aggregated with lipid II in the membrane.
Scherer et al., 2015	*Bacillus subtilis*	Nisin inhomogeneously bound lipid II, forming aggregates that caused cell death.
Vega and Caparon, 2012	*Streptococcus pyogenes*	Human neutrophil peptide 1 (HNP-1) and polymyxin B (PxB) disrupted anionic Exportal domain and delocalized the SecA translocase.
Kandaswamy et al., 2013	*Enterococcus faecalis*	Human β-defensin 2 (hBD2) focally targeted cell surface and delocalized Sortase A (SrtA) and the SecA translocase.
Wenzel et al., 2014	*Bacillus subtilis*	A 6-amino acid CAMP delocalized the peripheral membrane proteins MurG and cytochrome c.
Zweytick et al., 2014	*E. coli*	Lactoferricin-derived N-acylated AMPs disrupted phosphatidylethanolamine (PE) and cardiolipin membrane domains and caused defective cell division.
Rangarajan et al., 2013	*E. coli*	Cecroprin A permeabilized the outer and cytoplasmic membranes in discrete membrane regions.
Tran et al., 2013	*Enterococcus faecalis*	Daptomycin targeted the division septum.
Pogliano et al., 2012	*Bacillus subtilis*	Daptomycin delocalized cell division protein DivIVA.
Rangarajan et al., 2013	*E. coli*	Cecroprin A caused localized permeabilization of the cell membrane

## Sensing and adaptation to CAMPs

In response to environmental stressors, including AMPs, bacteria sense and induce transcriptional responses through two-component regulatory systems (TCS). Most TCS have two main components: (1) a histidine kinase sensor protein located in the cell membrane, and (2) a response regulator in the cytoplasm. While the functions of TCS aren't limited to antimicrobial sensing and responses, here we will focus only on their functions in relation to CAMPs. One regulatory consequence of TCS sensing of CAMPs is the induction of genes involved in CAMP-resistance. Additional resistance mechanisms involve chemical modifications to structures in the cell envelope. A recent review summarizes the processes that promote resistance to AMPs as follows: (1) modifications to the bacterial surface of (a) Gram-positive bacteria, including extracellular polysaccharides, membrane phospholipids such as PG, alterations in fatty acid composition, alanylation of LTA, and (b) Gram-negative bacteria, including modification of the lipid A component of lipopolysaccharide (LPS) through acylation/deacylation, glycinylation, hydroxylation, and dephosphorylation; lipid A modification with aminoarabinose, glucosamine, or galactosamine; and modification of LPS O-antigen length; (2) peptide property changes resulting from binding to the bacterial surface; (3) biofilm-related resistance mechanisms; and (4) involvement of TCS (Nuri et al., 2015). Here we focus on recently discovered examples of surface modifications associated with CAMP resistance.

One of the most extensively studied TCSs that responds to CAMPs is the *Salmonella typhimurium* PhoPQ system (Galán and Curtiss, 1989; Miller et al., 1989; Groisman, 2001). A highly acidic region in PhoQ directly recognizes CAMPs (Bader et al., 2005), inducing PhoQ signaling to PhoP to regulate various CAMP resistance mechanisms, including lipid A remodeling and enhanced intracellular survival within acidified phagosomes (Dalebroux and Miller, 2014). More recently, PhoPQ was shown to increase the levels of palmitoylated acylphosphatidylglycerols and CL, both of which are glycerophospholipids (GPL) found in the inner leaflet of the outer membrane of this Gram-negative organism. The joint regulation of the GPLs and lipid A structures mediates CAMP resistance by altering hydrophobicity and surface charge of the outer membrane and limiting CAMP binding (Dalebroux et al., 2014; Matamouros and Miller, 2015). After activation, PhoQ induces a transmembrane protein (PbgA) to transfer CL from the inner membrane to the outer membrane. The globular region of PbgA binds CL near the inner membrane and mediates CL trafficking to the outer membrane. Mutants that lack this globular region are less virulent (Dalebroux et al., 2015). How increased CL levels increase CAMP resistance is not yet known. One possibility is that CL might undergo conversion to lysyl-cardiolipin (Geiger et al., 2010) once it has reached the outer membrane, similar to the lysinylation of PG. The net positive charge of lysyl-cardiolipin would repel CAMPs, focally or otherwise.

Similar to the lipid A remodeling that occurs in *Salmonella* to confer CAMP resistance, *A. baumanii* CAMP resistance results from the addition of palmitate to the R-2-hydroxymyristate of lipid A, increasing outer membrane hydrophobicity (Boll et al., 2015). Increased hydrophobicity would hinder the diffusion of CAMPs through the outer membrane. Lipid A palmitylation in *A. baumanii* occurs via two putative acyltransferases (LpxL_Ab_ and LpxM_Ab_) that mediate the respective addition of one and two lauryl (C_12:0_) chains during synthesis of lipid A, rendering the bacteria resistant to polymyxins (Boll et al., 2015). Lipid A remodeling also occurs in *Vibrio cholera* and is under the regulation of the VprAB TCS. This system becomes activated in response to CAMPs and brings about a lipid A glycine modification. When glycine or diglycine is added to lipid A, the surface charge becomes less negative, leading to electrostatic repulsion of CAMPs and polymyxin B (Herrera et al., 2014). *P. aeruginosa* strains that are resistant to polymyxin harbor mutations that map to a TCS that resembles the PmrAB TCS of *Salmonella enterica* serovar Typhimurium (Levy and Marshall, 2004; Moskowitz et al., 2004). PmrAB is activated by PhoPQ and both these systems are activated by CAMPs *in vitro* and *in vivo* (Richards et al., 2012). Polymyxin resistance was correlated with lipid A modification with aminoarabinose in *P. aeruginosa* (Moskowitz et al., 2004). In *K. pneumoniae*, both the PhoPQ and PmrAB TCSs were recently shown to be involved in the response to treatment with PxB. PxB exposure resulted in the upregulation of genes involved in the modification of lipid A with aminoarabinose and palmitate, as well as the capsule polysaccharide operon. These surface changes contributed to CAMP resistance. Cross-talk between PhoPQ and PmrAB two systems and the regulator of capsule synthesis (Rcs) system also occurs, but the exact signal for the sensor kinase, RcsC, remains unclear. Thus, the CAMP response mounted by *K. pneumoniae* is mediated by three TCS systems (Llobet et al., 2011). Since lipid A and phospholipids are co-regulated by PhoPQ in *S. enterica* serovar Typhimurium, the possibility that phospholipids are co-regulated with lipid A in *V. cholera* and *K. pneumoniae* is worth investigating.

**Figure 1:**
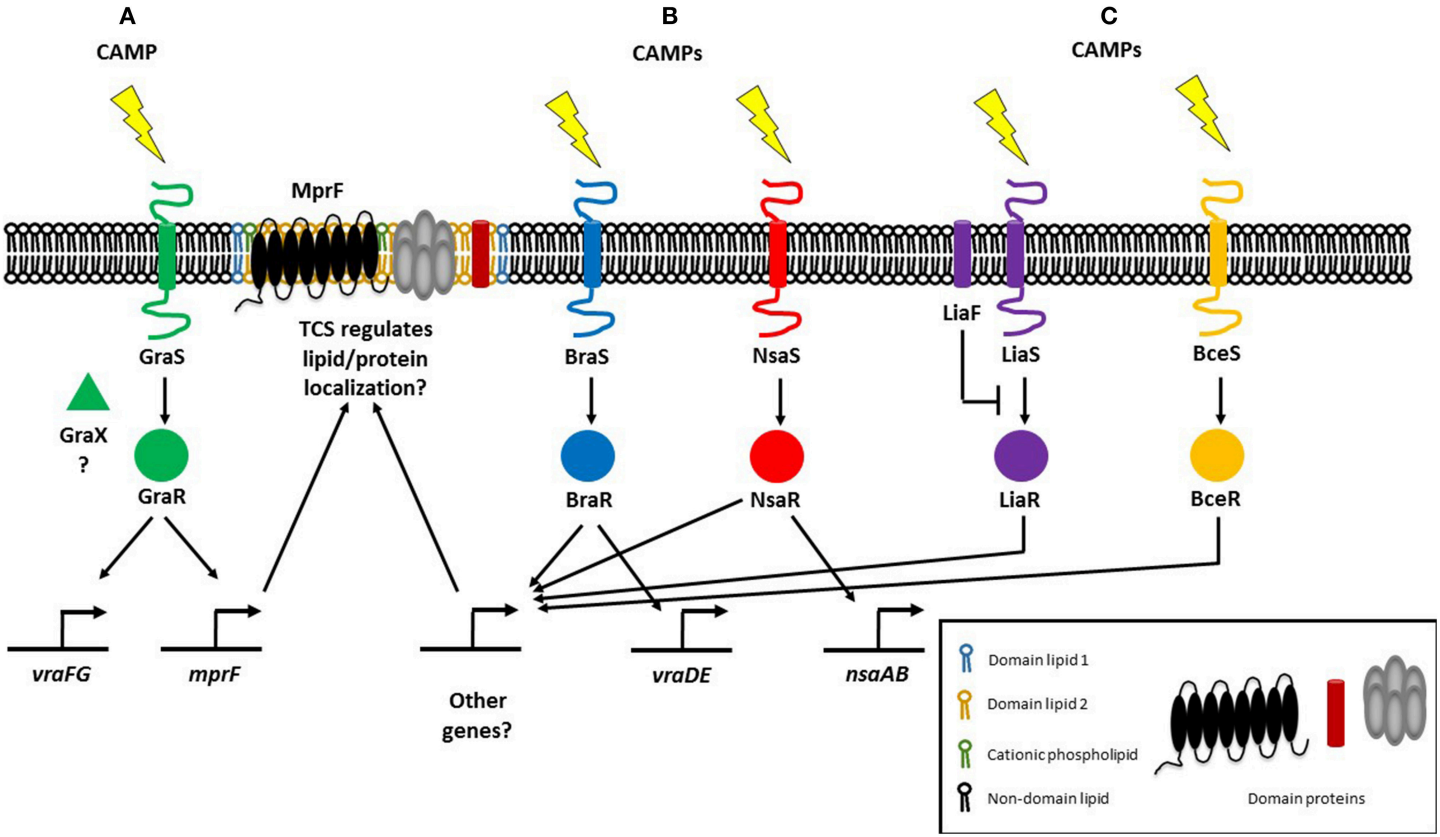
**Regulatory systems mediating resistance to cationic antimicrobial peptides (CAMPs) in Gram-positive bacteria. (A)** The three-component regulatory system GraRSX (also known as ApsRSX) regulates the expression of multiple peptide resistance factor (MprF), the bifunctional enzyme responsible for synthesizing and flipping cationic lysyl-phosphatidylglycerol to the outer membrane leaflet (Li et al., 2007a). **(B)** In addition to known target genes, the BraRS and NsaRS two-component regulatory systems may also regulate genes involved in lipid homeostasis (Kolar et al., 2011). **(C)** *E. faecalis* possesses the LiaFSR TCS which responds to daptomycin and regulates cardiolipin (CL) septal localization (Tran et al., 2013). In *B. subtilis*, LiaRS detects CAMPs directly, whereas BceRS detects CAMPs indirectly (Wolf et al., 2012).

CAMP resistance in Gram-positive bacteria is similarly associated with lipid modifications that alter their net surface charge. In Staphylococci, CAMPs are sensed by a three-component regulatory system known as ApsRSX. ApsRSX was discovered in *Staphycoccus epidermidis* and was the first CAMP sensing system to be reported for Gram-positive bacteria (Li et al., 2007b). The system comprises a classical TCS where ApsS is the histidine kinase sensor and ApsR is the DNA-binding response regulator. The function of the third component, ApsX, is still ambiguous but ApsX is unique to the staphylococci (Li et al., 2007b). The ApsR-ApsS TCS and regulatory cofactor ApsX are also known as GraR-GraS and GraX and have been associated with CAMP resistance but also with high temperature and oxidative stress responses, as well as in pathogenesis (Falord et al., 2011; Joo and Otto, 2015). The expression of MprF is under the control of GraRS, as is a putative AMP transport system encoded by *vraFG* (Li et al., 2007a; Figure 1A). Alterations in phospholipid composition only have minor correlations with phospholipid synthesis gene expression (Kuhn et al., 2015). Staphylococci have additional TCSs that mediate CAMP resistance via other mechanisms, notably increased efflux. The BraRS and NsaRS systems in *S. aureus* are essential for resistance to several CAMPs including nisin and bacitracin (Pietiainen et al., 2009; Kolar et al., 2011; Figure 1B). The *braRS* genes are present in the genome upstream of *braDE* (an ABC-type transporter system), and also activate another ABC transporter operon, *vraDE* (Kolar et al., 2011). Additionally, NsaAB is regulated by NsaRS, a TCS which senses damage to the cell envelope (Kolar et al., 2011). It remains to be seen whether any of these other TCSs regulate the localization of membrane lipids and/or proteins, although NsaRS deletion mutants show a more diffuse cell wall and increased encapsulation (Kolar et al., 2011; Figure 1B).

Gram-positive *E. faecalis* also regulates membrane phospholipids in response to CAMPs through a three-component regulatory system. *E. faecalis* possess the LiaFSR TCS that senses and responds to antibiotics and CAMPs (Figure 1C), a system that is also present in *Streptococcus mutans* and *B. subtilis* where it controls cell envelope homeostasis and regulates antibiotic and CAMP responses (Jordan et al., 2006; Suntharalingam et al., 2009; Kesel et al., 2013). In daptomycin-sensitive cells, CL microdomains are localized to the division septum. Single amino acid deletions in LiaF, GdpD, and Cls, which are thought to be gain-of-function mutations, result in CL redistribution away from the septum. Deletion of *liaR* which encodes the LiaFSR response regulator, from the mutant carrying amino acid deletions in LiaF, GdpD, and Cls restores the septal localization of CL microdomains to that of the wild type daptomycin-sensitive strain. CL microdomain localization is thus determined by LiaR (Reyes et al., 2015). Previously, a *liaF* mutation affecting septal binding of daptomycin was accompanied by mutations in glycerophosphoryldiester-phosphodiesterase *(gdpD)* and cardiolipin synthase *(cls)*, levels of PG and CL decreased whereas the level of glycerophosphoryl diglucosyl diacylglycerol increased, conferring full resistance to daptomycin (Tran et al., 2013). Thus, the LiaFSR system initiates antimicrobial resistance by changing cardiolipin localization and further augments resistance by with the help of *gdpD* and *cls*, which are both involved in lipid metabolism. LiaRS involvement in CAMP resistance is not restricted to the Enterococci, although it remains to be seen whether lipid metabolism and/or membrane localization are controlled by LiaRS in other organisms. In *B. subtilis*, while both LiaRS and BceRS respond to antimicrobial challenges at the membrane, their roles vary: LiaRS senses and responds to CAMPs indirectly, while the BceRS module detects CAMPs directly (Wolf et al., 2012; Figure 1C). The LiaRS role as damage sensor has also been demonstrated in the corresponding system in *Streptococcus pneumoniae* (Eldholm et al., 2010). LiaR also mediates resistance to nisin in *Listeria monocytogenes* (Bergholz et al., 2013).

Anionic phospholipid headgroup modifications render the hydrophilic moiety of phospholipids positive, leading to CAMP electrostatic repulsion, and resistance. However, the fatty acyl chains that make up the hydrophobic moiety of a phospholipid also facilitate CAMP resistance. Resistance to the CAMP pediocin in *E. faecalis* was recently reported to be due to a reduction in the proportion of branched chain fatty acids in the membrane, resulting in increased membrane rigidity that impeded pediocin penetration and pore formation. In addition, resistance to the CAMP pediocin was associated with increased expression of *mprF*, which functions to conjugate the amino acid lysine to PG. Thus, pediocin resistance was also correlated with a higher surface positive charge (Kumariya et al., 2015). Pediocin-resistant *E. faecalis* strains displayed cross-resistance to human neutrophil peptide 1 (HNP-1), nisin, and alamethicin (Kumariya et al., 2014). In contrast, increased membrane fluidity accompanied daptomycin resistance in *S. aureus* strains (Mishra et al., 2011; Mishra and Bayer, 2013). Whether lower or higher fluidity promotes CAMP resistance depends on the physical and chemical properties of the CAMP in question and its specific mechanism of action.

A widely conserved bacterial homolog of eukaryotic dynamin-like proteins (DLP), DynA, was recently shown to partially protect *B. subtilis* against antibiotics and phages (Sawant et al., 2015). This is a substantial new finding because apart from the ability of DLPs to re-model membranes *in vitro* (Low and Löwe, 2006; Bürmann et al., 2011), very little was known about their function in bacteria (Bramkamp, 2012). There is now evidence to suggest that DynA contributes to membrane protection against antibiotics and bacteriophages by closing membrane pores via membrane tethering and fusion (Bürmann et al., 2011; Sawant et al., 2015). It will be important to determine whether DynA plays a similar role in other bacteria and to determine whether it can be targeted as part of a novel therapeutic strategy that would inhibit its ability to repair membrane damage. It will also be interesting to see whether DynA activity is associated with changes in lipid levels that might assist in pore sealing.

**Figure 2:**
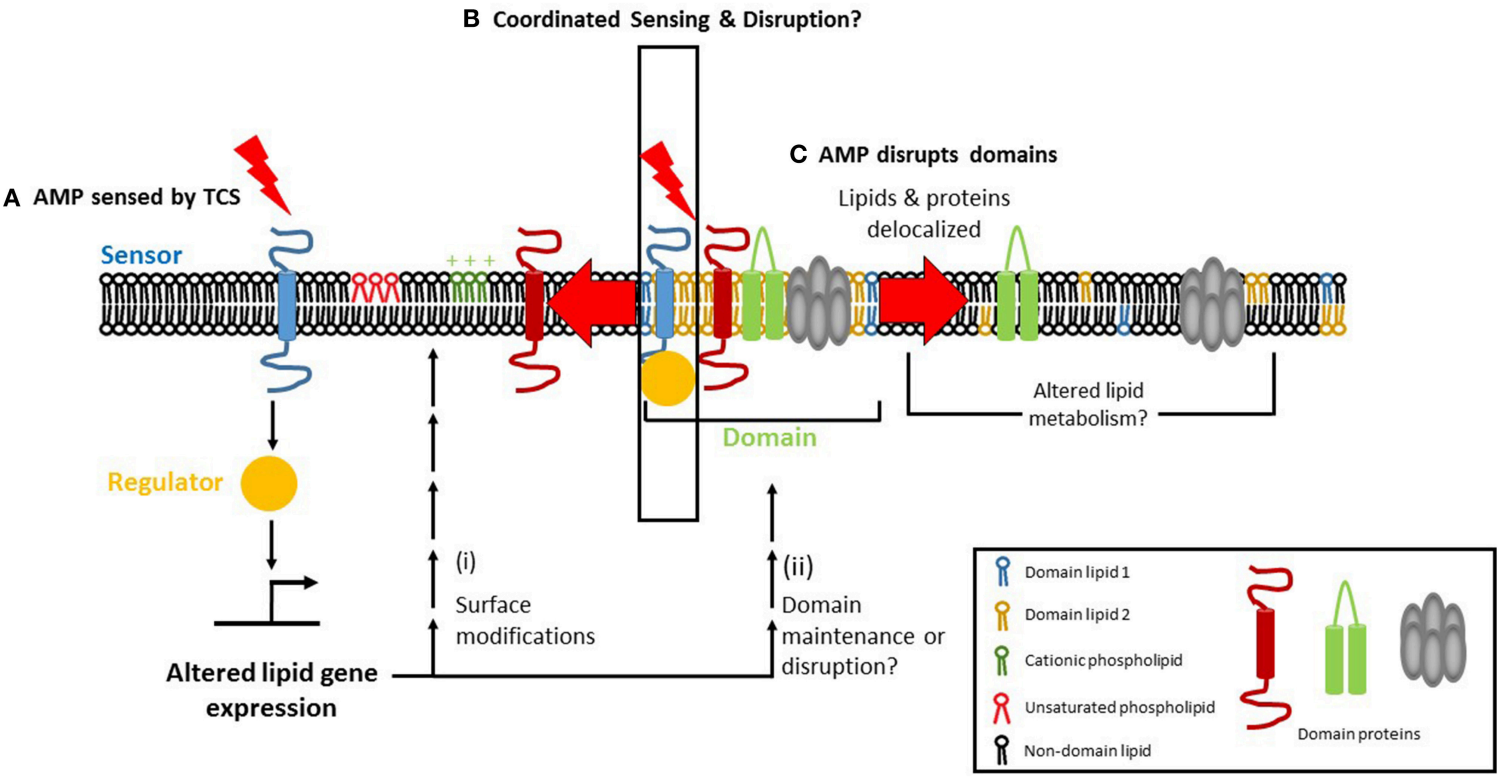
**Focal targeting of cationic antimicrobial peptides (CAMP) to the bacteria cell membrane and its consequences. (A)** Two-component regulatory systems (TCS) can sense CAMPs via their sensor histidine kinase. The sensor then activates a response regulator which can affect lipid homeostasis, either transcriptionally or post-transcriptionally, leading to (i) surface modification of phospholipid charge and/or degree of fatty acid saturation, alterations that mediate resistance to AMPs. (ii) In parallel, TCS activation may promote the maintenance of membrane domains (if present) or cause their disruption. **(B)** Membrane microdomains serve as focal targets for the CAMP, which causes delocalization of domain lipids (1 and 2 depict the unique lipids found in domains) and/or proteins upon binding. Lipid delocalization may alter the metabolism of lipids in a post-transcriptional manner, via phospholipid and fatty acid recycling pathways. **(C)** The TCS may itself be domain-localized, enabling coordinated AMP sensing, and disruption to occur.

The discovery that the PhoPQ of *S. enterica* serovar Typhimurium regulates membrane phospholipids raises the possibility that TCSs involved in CAMP resistance in other bacteria may also regulate phospholipids. In *E. faecalis*, a *liaF* mutation resulted in delocalization of CL microdomains and off-target daptomycin binding that corresponds with daptomycin resistance. The preference of CAMPs like Cecroprin A and LL-37 to bind to the septum and poles, where the cardiolipin content is high, suggests that these focal interactions might be carefully regulated by TCS genes, perhaps even in a localization-dependent manner. A model emerges in which CAMP exposure is sensed by a TCS within the membrane, leading to transcriptional changes that modify the membrane's composition which, in turn, leads to alterations in charge, fluidity and organization (Figure 2).

## Conclusions and future challenges

Research into the bacterial plasma membrane holds great promise for understanding the focal targeting of AMPs. Yet, there are a great number and variety of questions which are yet to be answered. The first step in studying the membrane is to determine the membrane lipid composition of any given bacterial species. This is achieved through the tools and expertise of the emerging field of lipidomics which is defined as the “system-level analysis and characterization of lipids and their interacting moieties” (Wenk, 2005). Lipidomics will enable us to appreciate the diversity of bacterial lipids, study similarities and differences in lipid composition across bacterial species, predict the types of domains that may be present in the membrane, and ultimately design new membrane-targeting antimicrobial compounds. Mass spectrometry (MS) combined with chromatographic separation is the backbone of lipidomics and this technology is developing rapidly to cater to the needs of the microbial research community (Wenk, 2010). A bacterial lipidomic workflow generally involves extracting lipids from cells grown either in planktonic or biofilm cultures, analysis by a suite of MS techniques that includes shotgun MS and LC-MS/MS, obtaining a lipid profile and identifying the lipids in that profile with the help of curated lipid databases, literature searches, and/or structural predictions. Once lipids have been identified, they can be quantified by multiple-reaction monitoring (MRM) (Shui et al., 2012). Using these methods, the charged and amphipathic phospholipids and their modified counterparts, as well as neutral lipids such as isoprenoid lipids and sterol-like lipids, may be characterized.

Lipidomic data will be helpful in predicting what types of domains may be present. Lipid domains are common structural features that promote the heterogeneous distribution of lipids in the plasma membrane of Gram-positive and Gram-negative bacteria and in the mycolate outer membrane of mycobacteria. However, the molecular details of the composition and assembly of these domains have yet to be completely elucidated and will require both lipidomics to determine lipid composition and imaging to observe domain location and track their spatial and temporal dynamics. Integrating this information will both improve our understanding of bacterial physiology and suggest new and effective ways of combatting bacterial pathogens. Techniques that have been used for studying bacterial lipid rafts include isolating detergent-resistant membranes (DRMs), fluorescence microscopy, depletion of lipid raft-associated proteins and/or lipids, and the inhibition of protein or lipid synthesis (Farnoud et al., 2015). To track the dynamics of lipid rafts or any other domain type, fluorescence correlation spectroscopy (FCS) and fluorescence recovery after photobleaching (FRAP) may be used to study the lateral diffusion of membrane proteins (Chow et al., 2012). Super-resolution microscopy may also be used to observe lipid rafts.

The time resolution of conventional biochemical assays for studying the destructive effects of CAMPs, such as membrane depolarization, cell lysis, and killing, is on the order of minutes to hours, which is not sufficient for capturing the mechanism of rapid-acting agents. In addition to poor time resolution, these assays cannot detect inter-cell heterogeneity. Studies on CAMP mechanisms will be greatly enhanced by the high spatiotemporal resolution offered by single-cell, time-resolved imaging assays (Choi et al., 2016b). Time-resolved imaging studies allowed the mechanism of action of a highly cationic random nylon-3 co-polymer to be elucidated (Choi et al., 2016a). The co-polymer rapidly traversed the outer membrane within seconds. As a result of co-polymer entry, bubbles formed in the periplasm, which led to cell shrinkage and growth cessation. Permeabilization of the cytoplasmic membrane then ensued in a spatially localized manner. Unlike CAMPs, the cationic co-polymer could traverse the outer membrane within seconds and efforts are currently underway to determine which polymer properties facilitate this rapid translocation (Choi et al., 2016a). High-resolution imaging techniques will enable a more complete understanding of how CAMPs and membrane permeabilizing agents in general act on bacteria in both space and time.

Another question related to domain research is whether cytoskeletal proteins affect domain formation. A new technique for studying membrane domains is nanoscale secondary ion mass spectrometry (NanoSIM). The main advantage of using this imaging mass spectrometry-based approach is that it is label-free, unlike fluorescence microscopy which requires a fluorescent label to be added to or expressed by bacterial cells. Lipid quantification may also be carried out if required. NanoSIM has been used on artificial lipid bilayers (Lozano et al., 2013) and mouse fibroblast cells (Frisz et al., 2013). For bacteria, NanoSIM has was used to visualize domains containing hopanoid lipids in *Rhodopseudomonas palustris* TIE-1 and *Nostoc punctiforme* PCC73102 (Doughty et al., 2014). Hopanoids function to increase the rigidity of the plasma membrane at high temperature (Sáenz et al., 2012) and they may also influence CAMP targeting by changing membrane fluidity within domains. NanoSIM holds great promise for visualizing membrane domains in various bacterial species.

In addition to traditional DRM fractionation to isolate lipid rafts, a new, alternative approach employs styrene-maleic acid (SMA) copolymer which, after inserting itself into the membrane, is capable of extracting nano-sized discs that contains proteins and lipids (Dörr et al., 2016). SMA was used to isolate and characterize the tetrameric potassium ion channel KcsA from *E. coli* (Dörr et al., 2014). KcsA was thermally more stable in the native nanodiscs than in detergent micelles (Dörr et al., 2014). SMA nanodiscs have been used to extract a variety of proteins from bacterial, yeast, and human cells (Dörr et al., 2016). Nanodiscs are likely to be useful in studying CAMP effects on domain structure and function in the future.

Another level of membrane heterogeneity is the recently described phenomenon of “transertion” (Fishov and Norris, 2012; Matsumoto et al., 2015). The sources of heterogeneity in the plasma membrane are lipid-lipid, lipid-protein, and protein-protein interactions. When these interactions become physically associated with transcription, translation, and nascent protein insertion into the membrane, large membrane domains are formed. This process of transertion has been described as a global regulator that couples cell metabolism to the cell cycle (Fishov and Norris, 2012). It is an example of how membrane domains guide the spatial and temporal organization of bacteria. Future studies could explore a possible connection between these specialized domains and CAMP action. CAMPs might physically disrupt the domains or they might interfere with transertion domain-related processes, e.g., transcription, translation, lipid-protein interactions, or protein-protein interactions.

Lipidomics can be used to study of the role of phospholipid variants called plasmalogens in CAMP resistance. Plasmalogens are found in organisms ranging from bacteria to mammals (Rezanka et al., 2012) and were first detected in anaerobic bacteria such as Ruminococci (Allison et al., 1962) and *Clostridium butyricum* (Goldfine, 1964) five decades ago. In the structure of plasmalogen, one fatty acid chain is connected to glycerol by a ester linkage while the other fatty acid is connected by a vinyl ether linkage (Rezanka et al., 2012). Plasmalogens provide protection against acidic or alkaline pH, high temperature, organic solvents, and antibiotics (Lee et al., 1998). It was reported recently that the plasmalogen content of *Clostridium pasteurianum* rose in the presence of excess butanol (Kolek et al., 2015). It will be interesting to see how widely expressed this type of phospholipid is amongst anaerobic pathogens, and liquid chromatography-mass spectrometry would be the technique of choice for identifying and quantifying plasmalogens.

A technique which is particularly amenable to studying the role of lipids in processes such as bacterial cell division and secretion is metabolic labeling via bio-orthogonal chemistry (Siegrist et al., 2015). Lipids can be labeled *in situ* and their spatio-temporal dynamics can be tracked as cells grow, divide, and secrete. Metabolic labeling is minimally invasive and macromolecules can be studied in their natural states. Bio-orthogonal chemistry has been used to study post-translational modifications in the mammalian HeLa cell line (Grammel et al., 2011), and has been reviewed elsewhere (Grammel and Hang, 2013). Bio-orthogonal chemistry provides an alternative to using radioactive labels, and involves a chemical substrate which is incorporated into a macromolecular structure and is rendered detectable by a fluorophore or affinity tag, which becomes covalently linked to the macromolecule of interest (Patterson et al., 2014; Siegrist et al., 2015). This technique could be used to study the dynamics of membrane domains and interactions between membrane lipids and proteins, as well as the effects of membrane curvature, membrane potential, pH, temperature, osmolarity, and AMPs on the aforementioned processes.

How do bacteria achieve homeostasis of the plasma membrane in response to CAMPs? The lipidome of a cell is a reflection of the specific environmental conditions that it has recently encountered. External factors such as osmolarity, temperature, and pH can affect lipid composition. It is already known, for example, the DesK-DesR TCS increases fatty acid unsaturation at cold temperatures in *B. subtilis* (Aguilar et al., 2001). What is yet to be determined is how the change in lipid composition resulting from the change in temperature is sensed by DesK. If DesK does not sense temperature directly or in isolation, then what are the other players? A recent review summarizes factors that must be considered for the study of sensor histidine kinases (Puth et al., 2015). The sensor histidine kinase of a TCS senses a CAMP and activates its cognate response regulator, which transcriptionally or post-transcriptionally produces membrane lipid modifications that confer CAMP resistance (Figure 2A). Correlating lipidomic changes with transcriptomic and proteomic data will help identify the bacterial factors involved in regulating membrane lipid composition and/or domain structure. In addition, changes in the lipid profiles of bacteria that have been treated with antibiotics or AMPs can be combined with data about the TCS involved in responding to antimicrobial stress, thus providing information about the mechanisms underlying the response. This approach will lend new insights and suggest ways to circumvent bacterial adaptation and resistance. KinC, a sensor histidine kinase involved in biofilm formation, was detected in DRM fractions of *B. subtilis* and *S. aureus*, suggesting that KinC is domain-localized (López and Kolter, 2010). It will be interesting to see whether other sensor histidine kinases are domain-localized and whether domain-localized TCSs occur in other bacteria. A domain-localized TCS may help to coordinate AMP sensing with cellular responses, which would result in a more rapid and effective response (Figure 2B). If TCS domain localization is beneficial for the cell, it might play a role in promoting AMP resistance. However, it is equally possible that such a benefit may be outweighed by the AMP's ability to disrupt the domain (Figure 2C). In short, these approaches will allow for the elucidation of how two-component systems operate at the molecular level.

Efforts are underway to develop novel CAMPs that circumvent known resistance mechanisms. Since most mechanisms depend on recognition of specific CAMP sequences or secondary structures, it is possible that minor alterations in these areas could restore or amplify their activity (Peschel and Sahl, 2006). Altering a peptide's sequence or structure to enhance activity presents challenges as care must be taken not only to retain activity against bacteria, but to remain non-toxic to host cells. Recent research suggests that the linear bumblebee peptides hymenoptaecin and abaecin may be successful in combination in killing Gram-negative bacterial pathogens (Rahnamaeian et al., 2015). A novel CAMP, T9W, has been found to be non-toxic to macrophages, synergistic with ciprofloxacin and gentamicin, and successful at disrupting the *P. aeruginosa* cell membrane (Zhu et al., 2015). Other compounds are targeting membrane biogenesis through via β-barrel protein LptD (Imp/OstA), a cellular protein target in Gram-negative bacteria (Srinivas et al., 2010) instead of the membrane itself. Another method might be to target the ribosome, as several novel compounds are doing. Short proline-rich antimicrobial peptides (PrAMPs) are a promising class of antimicrobials that bind to the bacterial 70S ribosome, inhibiting translation in *E. coli* (Krizsan et al., 2015) and *Thermus thermophilus* (Seefeldt et al., 2015). These new antimicrobials may not be foolproof either, though, as ribosomal targeting by classical antibiotics such as tetracycline is readily overcome by various pathogens (Schnappinger and Hillen, 1996).

In conclusion, here we have reviewed recent literature about the bacterial membrane, how AMPs can focally target the membrane, and the role of the membrane in sensing and responding to CAMP stress. Focal targeting of the bacterial envelope by CAMPs is a rapidly growing field and technological advances will help us to address many unanswered questions about how CAMPs delocalize lipids and proteins. Determining the lipid and protein composition of the domains targeted by CAMPs will reveal the targeting and disruption mechanisms. We expect that CAMP mechanisms of action will depend on several lipid-associated factors, including variations in phospholipid levels, the presence of positively-charged phospholipids such as lysyl-PG, the ratio of unsaturated fatty acids to saturated fatty acids in phospholipid molecules, and the presence of non-phospholipid lipids such as the isoprenoids. These factors will influence CAMP focal targeting, membrane binding and perturbation. In turn, bacteria may evolve CAMP resistance by modifying one or more of the above lipid-associated factors. To reveal domain-specific lipid modifications, lipidomics will need to be coupled to techniques that isolate membrane domains, such as detergent-independent domain isolation using the SMA co-polymer. Lipidomics will thus allow for the interrogation of these properties of the cell membrane. The envelope is all that separates the cytoplasm from the outside world with all its threats to cell survival. As we learn more about this crucial membrane structure, we will better understand how it enables bacteria to thrive in the vast varieties of environments they find themselves in and how they defend against the onslaught of extracellular stress and antimicrobials.

## Author contributions

All authors listed, have made substantial, direct and intellectual contribution to the work, and approved it for publication.

### Conflict of interest statement

The authors declare that the research was conducted in the absence of any commercial or financial relationships that could be construed as a potential conflict of interest.
